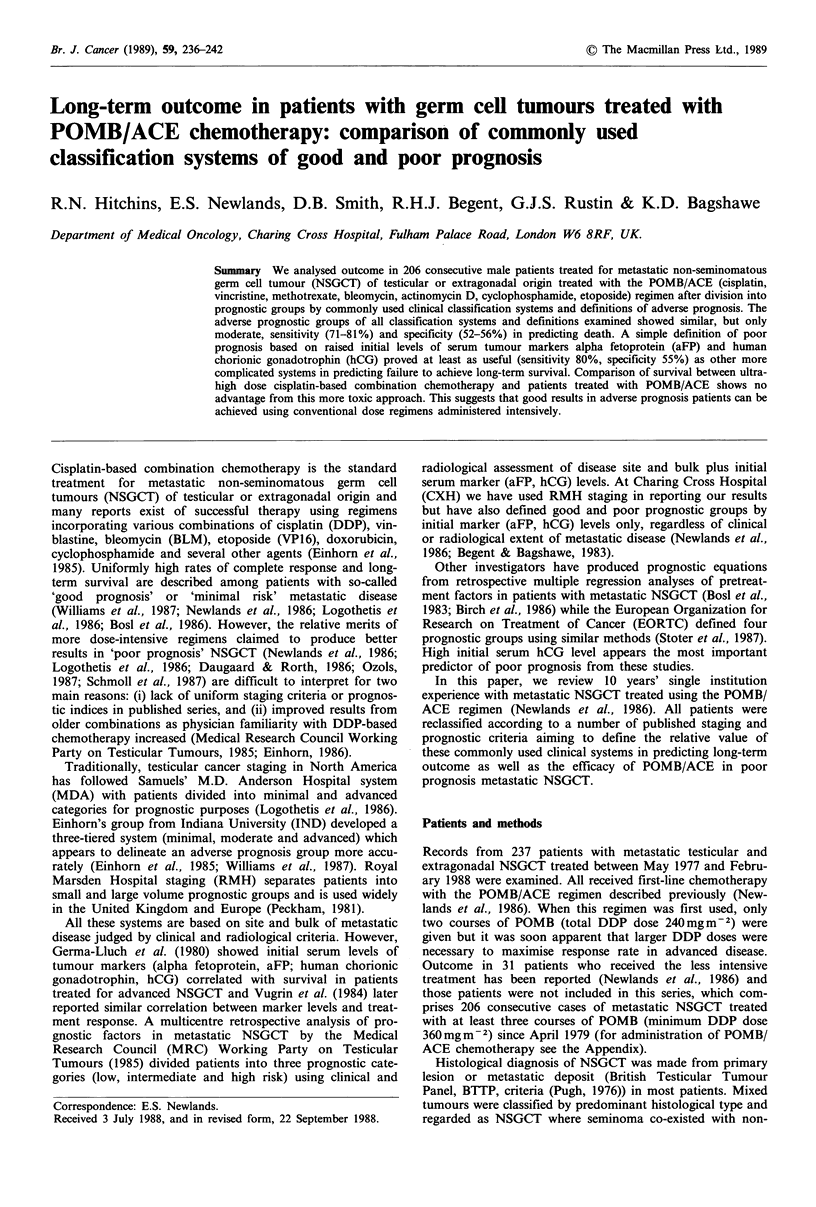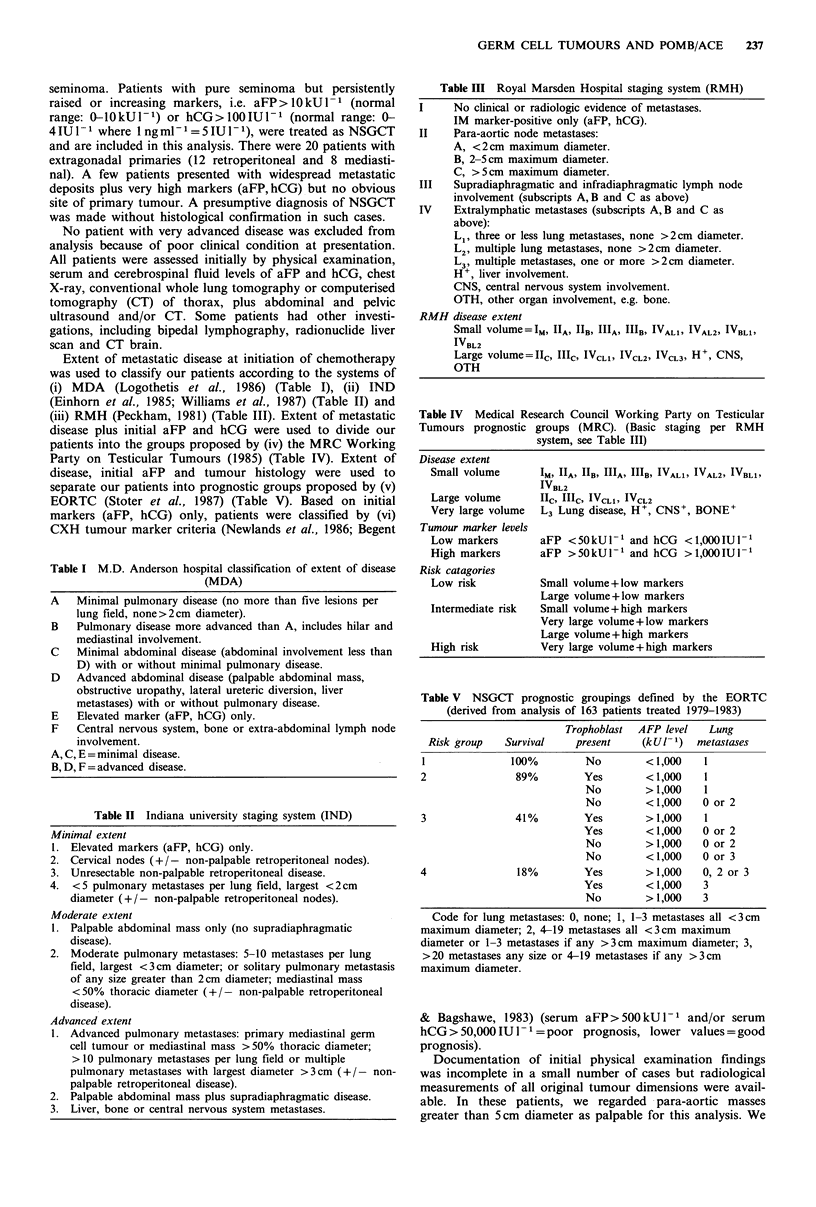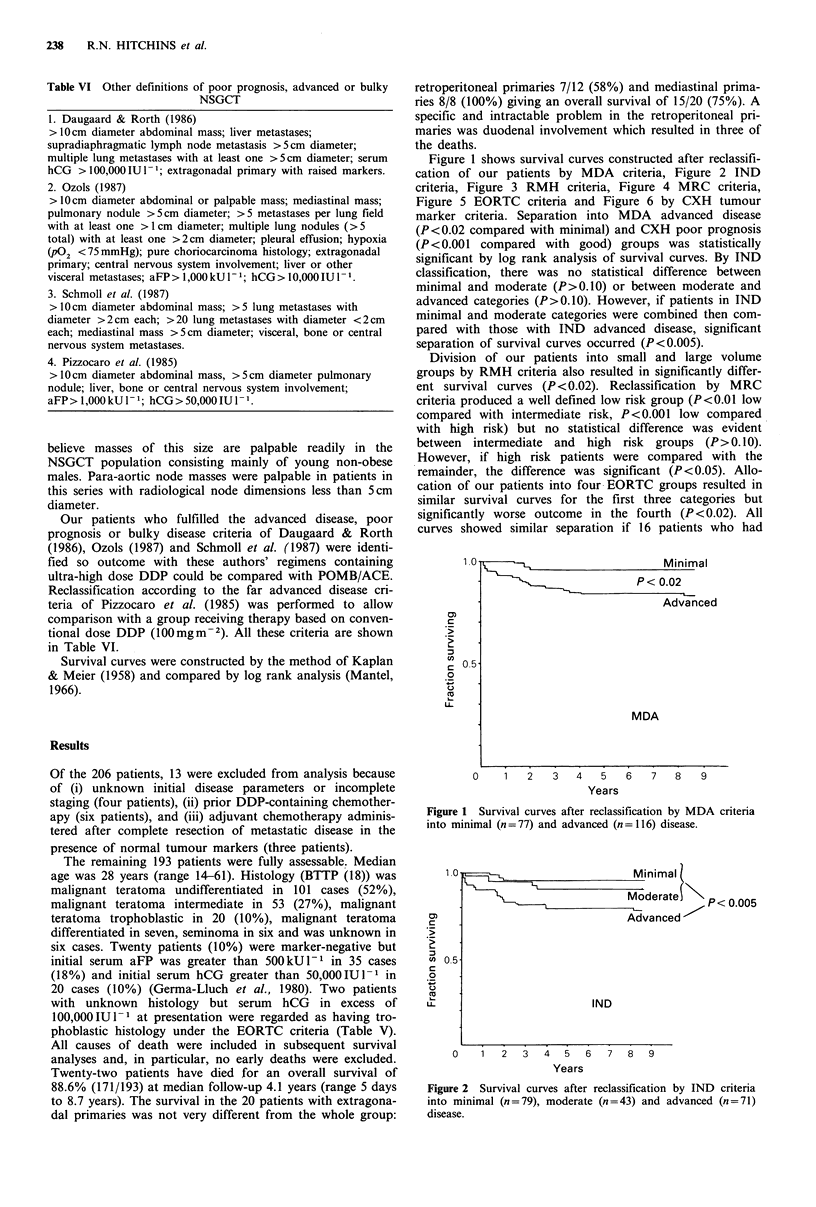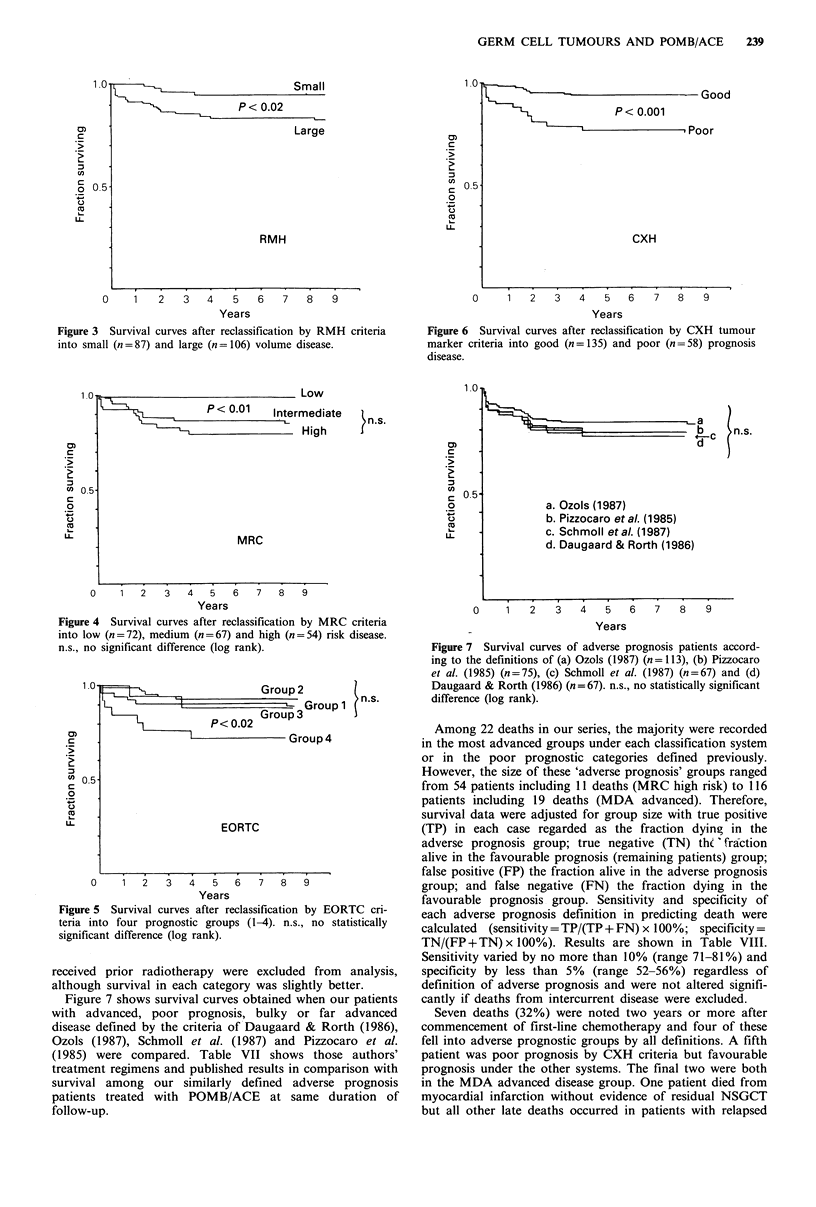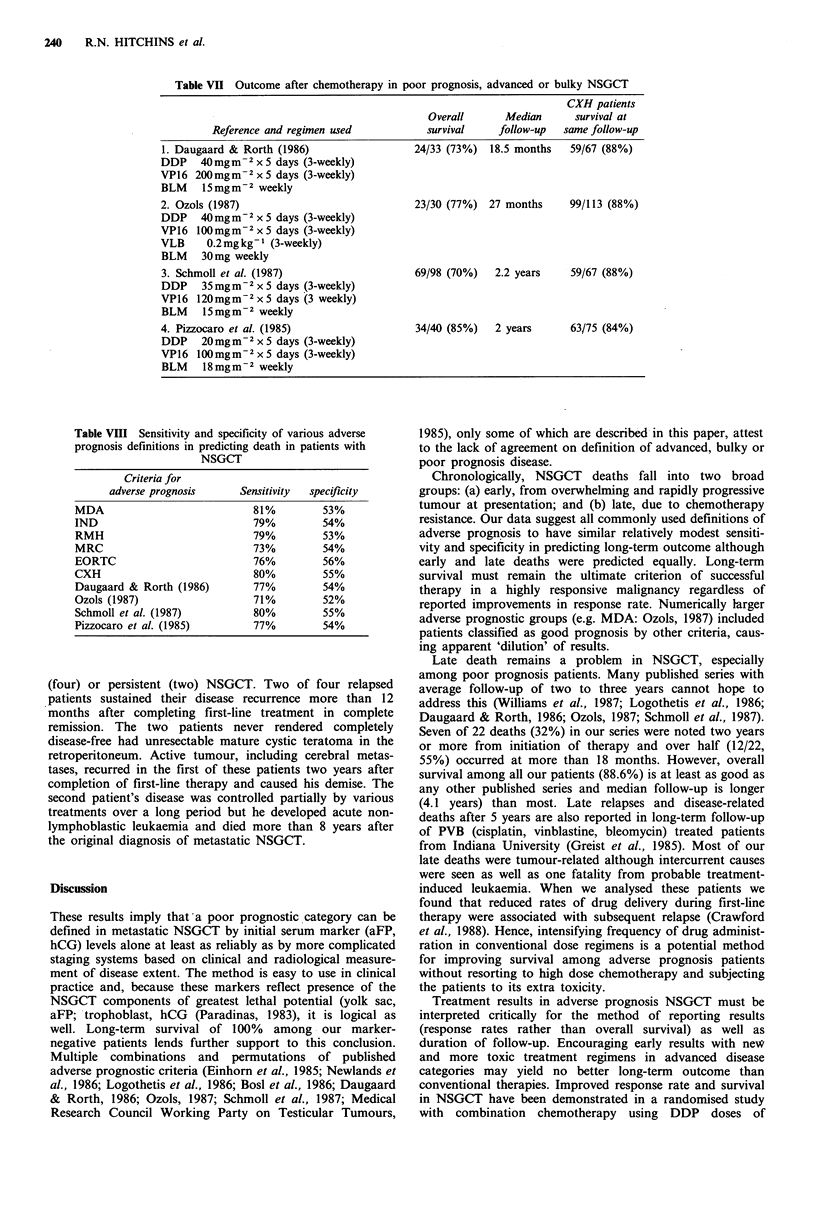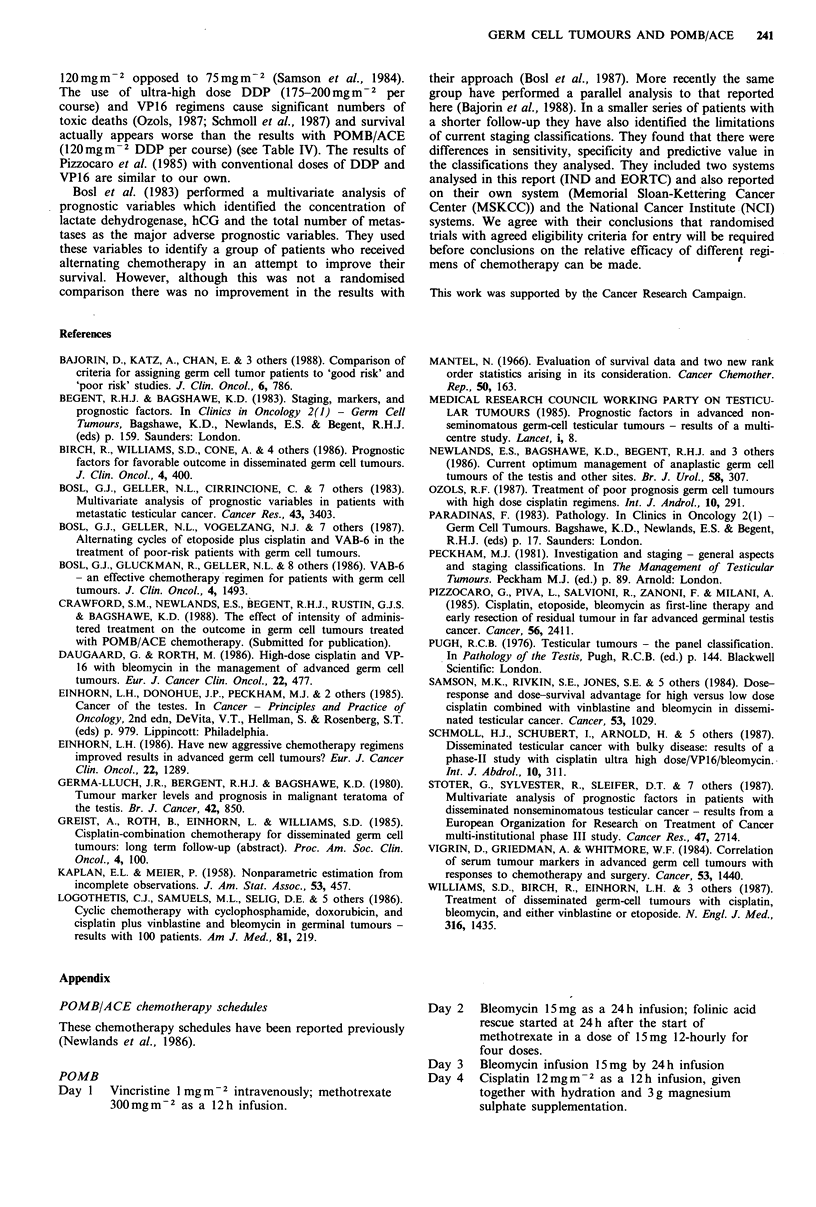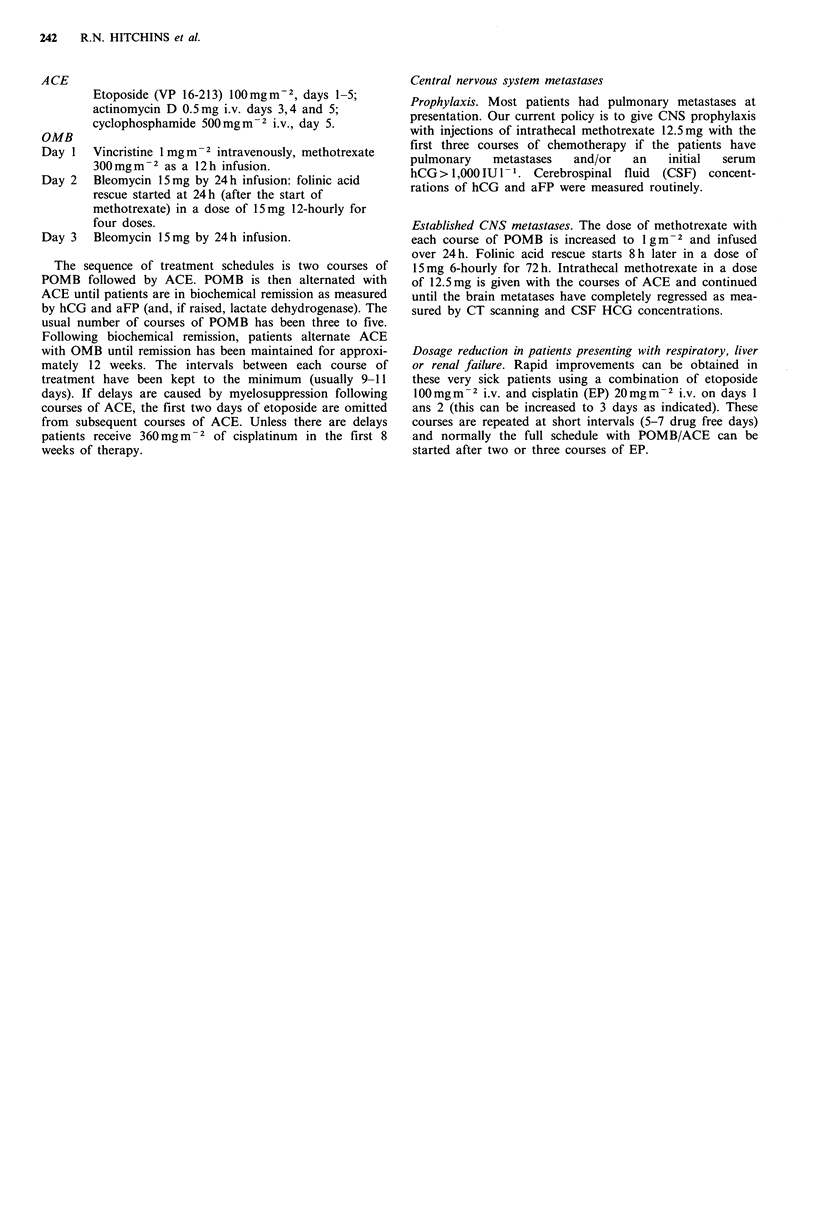# Long-term outcome in patients with germ cell tumours treated with POMB/ACE chemotherapy: comparison of commonly used classification systems of good and poor prognosis.

**DOI:** 10.1038/bjc.1989.48

**Published:** 1989-02

**Authors:** R. N. Hitchins, E. S. Newlands, D. B. Smith, R. H. Begent, G. J. Rustin, K. D. Bagshawe

**Affiliations:** Department of Medical Oncology, Charing Cross Hospital, London, UK.

## Abstract

We analysed outcome in 206 consecutive male patients treated for metastatic non-seminomatous germ cell tumour (NSGCT) of testicular or extragonadal origin treated with the POMB/ACE (cisplatin, vincristine, methotrexate, bleomycin, actinomycin D, cyclophosphamide, etoposide) regimen after division into prognostic groups by commonly used clinical classification systems and definitions of adverse prognosis. The adverse prognostic groups of all classification systems and definitions examined showed similar, but only moderate, sensitivity (71-81%) and specificity (52-56%) in predicting death. A simple definition of poor prognosis based on raised initial levels of serum tumour markers alpha fetoprotein (aFP) and human chorionic gonadotrophin (hCG) proved at least as useful (sensitivity 80%, specificity 55%) as other more complicated systems in predicting failure to achieve long-term survival. Comparison of survival between ultra-high dose cisplatin-based combination chemotherapy and patients treated with POMB/ACE shows no advantage from this more toxic approach. This suggests that good results in adverse prognosis patients can be achieved using conventional dose regimens administered intensively.


*Br. J. Cancer* (1989), **59**, 236–242 © The Macmillan Press Ltd., 1989

### Long-term outcome in patients with germ cell tumours treated with POMB/ACE chemotherapy: comparison of commonly used classification systems of good and poor prognosis

R.N. Hitchins, E.S. Newlands, D.B. Smith, R.H.J. Begent, G.J.S. Rustin & K.D. Bagshawe

*Department of Medical Oncology, Charing Cross Hospital, Fulham Palace Road, London W6 8RF, UK.*

**Summary** We analysed outcome in 206 consecutive male patients treated for metastatic non-seminomatous germ cell tumour (NSGCT) of testicular or extragonadal origin treated with the POMB/ACE (cisplatin, vincristine, methotrexate, bleomycin, actinomycin D, cyclophosphamide, etoposide) regimen after division into prognostic groups by commonly used clinical classification systems and definitions of adverse prognosis. The adverse prognostic groups of all classification systems and definitions examined showed similar, but only moderate, sensitivity (71–81%) and specificity (52–56%) in predicting death. A simple definition of poor prognosis based on raised initial levels of serum tumour markers alpha fetoprotein (aFP) and human chorionic gonadotrophin (hCG) proved at least as useful (sensitivity 80%, specificity 55%) as other more complicated systems in predicting failure to achieve long-term survival. Comparison of survival between ultra-high dose cisplatin-based combination chemotherapy and patients treated with POMB/ACE shows no advantage from this more toxic approach. This suggests that good results in adverse prognosis patients can be achieved using conventional dose regimens administered intensively.

Cisplatin-based combination chemotherapy is the standard treatment for metastatic non-seminomatous germ cell tumours (NSGCT) of testicular or extragonadal origin and many reports exist of successful therapy using regimens incorporating various combinations of cisplatin (DDP), vinblastine, bleomycin (BLM), etoposide (VP16), doxorubicin, cyclophosphamide and several other agents (Einhorn *et al.*, 1985). Uniformly high rates of complete response and long-term survival are described among patients with so-called 'good prognosis' or 'minimal risk' metastatic disease (Williams *et al.*, 1987; Newlands *et al.*, 1986; Logothetis *et al.*, 1986; Bosl *et al.*, 1986). However, the relative merits of more dose-intensive regimens claimed to produce better results in 'poor prognosis' NSGCT (Newlands *et al.*, 1986; Logothetis *et al.*, 1986; Daugaard & Rorth, 1986; Ozols, 1987; Schmoll *et al.*, 1987) are difficult to interpret for two main reasons: (i) lack of uniform staging criteria or prognostic indices in published series, and (ii) improved results from older combinations as physician familiarity with DDP-based chemotherapy increased (Medical Research Council Working Party on Testicular Tumours, 1985; Einhorn, 1986).

Traditionally, testicular cancer staging in North America has followed Samuels' M.D. Anderson Hospital system (MDA) with patients divided into minimal and advanced categories for prognostic purposes (Logothetis *et al.*, 1986). Einhorn's group from Indiana University (IND) developed a three-tiered system (minimal, moderate and advanced) which appears to delineate an adverse prognosis group more accurately (Einhorn *et al.*, 1985; Williams *et al.*, 1987). Royal Marsden Hospital staging (RMH) separates patients into small and large volume prognostic groups and is used widely in the United Kingdom and Europe (Peckham, 1981).

All these systems are based on site and bulk of metastatic disease judged by clinical and radiological criteria. However, Germa-Lluch *et al.* (1980) showed initial serum levels of tumour markers (alpha fetoprotein, aFP; human chorionic gonadotrophin, hCG) correlated with survival in patients treated for advanced NSGCT and Vugrin *et al.* (1984) later reported similar correlation between marker levels and treatment response. A multicentre retrospective analysis of prognostic factors in metastatic NSGCT by the Medical Research Council (MRC) Working Party on Testicular Tumours (1985) divided patients into three prognostic categories (low, intermediate and high risk) using clinical and radiological assessment of disease site and bulk plus initial serum marker (aFP, hCG) levels. At Charing Cross Hospital (CXH) we have used RMH staging in reporting our results but have also defined good and poor prognostic groups by initial marker (aFP, hCG) levels only, regardless of clinical or radiological extent of metastatic disease (Newlands *et al.*, 1986; Begent & Bagshawe, 1983).

Other investigators have produced prognostic equations from retrospective multiple regression analyses of pretreatment factors in patients with metastatic NSGCT (Bosl *et al.*, 1983; Birch *et al.*, 1986) while the European Organization for Research on Treatment of Cancer (EORTC) defined four prognostic groups using similar methods (Stoter *et al.*, 1987). High initial serum hCG level appears the most important predictor of poor prognosis from these studies.

In this paper, we review 10 years' single institution experience with metastatic NSGCT treated using the POMB/ACE regimen (Newlands *et al.*, 1986). All patients were reclassified according to a number of published staging and prognostic criteria aiming to define the relative value of these commonly used clinical systems in predicting long-term outcome as well as the efficacy of POMB/ACE in poor prognosis metastatic NSGCT.

### Patients and methods

Records from 237 patients with metastatic testicular and extragonadal NSGCT treated between May 1977 and February 1988 were examined. All received first-line chemotherapy with the POMB/ACE regimen described previously (Newlands *et al.*, 1986). When this regimen was first used, only two courses of POMB (total DDP dose 240 mg m$^{-2}$) were given but it was soon apparent that larger DDP doses were necessary to maximise response rate in advanced disease. Outcome in 31 patients who received the less intensive treatment has been reported (Newlands *et al.*, 1986) and those patients were not included in this series, which comprises 206 consecutive cases of metastatic NSGCT treated with at least three courses of POMB (minimum DDP dose 360 mg m$^{-2}$) since April 1979 (for administration of POMB/ACE chemotherapy see the Appendix).

Histological diagnosis of NSGCT was made from primary lesion or metastatic deposit (British Testicular Tumour Panel, BTTP, criteria (Pugh, 1976)) in most patients. Mixed tumours were classified by predominant histological type and regarded as NSGCT where seminoma co-existed with non-

Correspondence: E.S. Newlands.
Received 3 July 1988, and in revised form, 22 September 1988.

seminoma. Patients with pure seminoma but persistently raised or increasing markers, i.e. aFP > $10\,kU\,l^{-1}$ (normal range: $0–10\,kU\,l^{-1}$) or hCG > $100\,IU\,l^{-1}$ (normal range: $0–4\,IU\,l^{-1}$ where $1\,ng\,ml^{-1} = 5\,IU\,l^{-1}$), were treated as NSGCT and are included in this analysis. There were 20 patients with extragonadal primaries (12 retroperitoneal and 8 mediastinal). A few patients presented with widespread metastatic deposits plus very high markers (aFP, hCG) but no obvious site of primary tumour. A presumptive diagnosis of NSGCT was made without histological confirmation in such cases.

No patient with very advanced disease was excluded from analysis because of poor clinical condition at presentation. All patients were assessed initially by physical examination, serum and cerebrospinal fluid levels of aFP and hCG, chest X-ray, conventional whole lung tomography or computerised tomography (CT) of thorax, plus abdominal and pelvic ultrasound and/or CT. Some patients had other investigations, including bipedal lymphography, radionuclide liver scan and CT brain.

Extent of metastatic disease at initiation of chemotherapy was used to classify our patients according to the systems of (i) MDA (Logothetis *et al.*, 1986) (Table I), (ii) IND (Einhorn *et al.*, 1985; Williams *et al.*, 1987) (Table II) and (iii) RMH (Peckham, 1981) (Table III). Extent of metastatic disease plus initial aFP and hCG were used to divide our patients into the groups proposed by (iv) the MRC Working Party on Testicular Tumours (1985) (Table IV). Extent of disease, initial aFP and tumour histology were used to separate our patients into prognostic groups proposed by (v) EORTC (Stoter *et al.*, 1987) (Table V). Based on initial markers (aFP, hCG) only, patients were classified by (vi) CXH tumour marker criteria (Newlands *et al.*, 1986; Begent & Bagshawe, 1983) (serum aFP > $500\,kU\,l^{-1}$ and/or serum hCG > $50{,}000\,IU\,l^{-1}$ = poor prognosis, lower values = good prognosis).

Documentation of initial physical examination findings was incomplete in a small number of cases but radiological measurements of all original tumour dimensions were available. In these patients, we regarded para-aortic masses greater than 5 cm diameter as palpable for this analysis. We

**Table I** M.D. Anderson hospital classification of extent of disease (MDA)

| | |
|---|---|
| A | Minimal pulmonary disease (no more than five lesions per lung field, none > 2 cm diameter). |
| B | Pulmonary disease more advanced than A, includes hilar and mediastinal involvement. |
| C | Minimal abdominal disease (abdominal involvement less than D) with or without minimal pulmonary disease. |
| D | Advanced abdominal disease (palpable abdominal mass, obstructive uropathy, lateral ureteric diversion, liver metastases) with or without pulmonary disease. |
| E | Elevated marker (aFP, hCG) only. |
| F | Central nervous system, bone or extra-abdominal lymph node involvement. |

A, C, E = minimal disease.
B, D, F = advanced disease.

**Table II** Indiana university staging system (IND)

*Minimal extent*

1. Elevated markers (aFP, hCG) only.
2. Cervical nodes (+/− non-palpable retroperitoneal nodes).
3. Unresectable non-palpable retroperitoneal disease.
4. < 5 pulmonary metastases per lung field, largest < 2 cm diameter (+/− non-palpable retroperitoneal nodes).

*Moderate extent*

1. Palpable abdominal mass only (no supradiaphragmatic disease).
2. Moderate pulmonary metastases: 5–10 metastases per lung field, largest < 3 cm diameter; or solitary pulmonary metastasis of any size greater than 2 cm diameter; mediastinal mass < 50% thoracic diameter (+/− non-palpable retroperitoneal disease).

*Advanced extent*

1. Advanced pulmonary metastases: primary mediastinal germ cell tumour or mediastinal mass > 50% thoracic diameter; > 10 pulmonary metastases per lung field or multiple pulmonary metastases with largest diameter > 3 cm (+/− non-palpable retroperitoneal disease).
2. Palpable abdominal mass plus supradiaphragmatic disease.
3. Liver, bone or central nervous system metastases.

**Table III** Royal Marsden Hospital staging system (RMH)

| | |
|---|---|
| I | No clinical or radiologic evidence of metastases. |
| | IM marker-positive only (aFP, hCG). |
| II | Para-aortic node metastases: |
| | A, < 2 cm maximum diameter. |
| | B, 2–5 cm maximum diameter. |
| | C, > 5 cm maximum diameter. |
| III | Supradiaphragmatic and infradiaphragmatic lymph node involvement (subscripts A, B and C as above) |
| IV | Extralymphatic metastases (subscripts A, B and C as above): |
| | $L_1$, three or less lung metastases, none > 2 cm diameter. |
| | $L_2$, multiple lung metastases, none > 2 cm diameter. |
| | $L_3$, multiple metastases, one or more > 2 cm diameter. |
| | $H^+$, liver involvement. |
| | CNS, central nervous system involvement. |
| | OTH, other organ involvement, e.g. bone. |
| *RMH disease extent* | |
| | Small volume = $I_M$, $II_A$, $II_B$, $III_A$, $III_B$, $IV_{AL1}$, $IV_{AL2}$, $IV_{BL1}$, $IV_{BL2}$ |
| | Large volume = $II_C$, $III_C$, $IV_{CL1}$, $IV_{CL2}$, $IV_{CL3}$, $H^+$, CNS, OTH |

**Table IV** Medical Research Council Working Party on Testicular Tumours prognostic groups (MRC). (Basic staging per RMH system, see Table III)

| | |
|---|---|
| *Disease extent* | |
| Small volume | $I_M$, $II_A$, $II_B$, $III_A$, $III_B$, $IV_{AL1}$, $IV_{AL2}$, $IV_{BL1}$, $IV_{BL2}$ |
| Large volume | $II_C$, $III_C$, $IV_{CL1}$, $IV_{CL2}$ |
| Very large volume | $L_3$ Lung disease, $H^+$, $CNS^+$, $BONE^+$ |
| *Tumour marker levels* | |
| Low markers | aFP < $50\,kU\,l^{-1}$ and hCG < $1{,}000\,IU\,l^{-1}$ |
| High markers | aFP > $50\,kU\,l^{-1}$ and hCG > $1{,}000\,IU\,l^{-1}$ |
| *Risk catagories* | |
| Low risk | Small volume + low markers |
| | Large volume + low markers |
| Intermediate risk | Small volume + high markers |
| | Very large volume + low markers |
| | Large volume + high markers |
| High risk | Very large volume + high markers |

**Table V** NSGCT prognostic groupings defined by the EORTC (derived from analysis of 163 patients treated 1979–1983)

| *Risk group* | *Survival* | *Trophoblast present* | *AFP level* ($kU\,l^{-1}$) | *Lung metastases* |
|---|---|---|---|---|
| 1 | 100% | No | < 1,000 | 1 |
| 2 | 89% | Yes | < 1,000 | 1 |
| | | No | > 1,000 | 1 |
| | | No | < 1,000 | 0 or 2 |
| 3 | 41% | Yes | > 1,000 | 1 |
| | | Yes | < 1,000 | 0 or 2 |
| | | No | > 1,000 | 0 or 2 |
| | | No | < 1,000 | 0 or 3 |
| 4 | 18% | Yes | > 1,000 | 0, 2 or 3 |
| | | Yes | < 1,000 | 3 |
| | | No | > 1,000 | 3 |

Code for lung metastases: 0, none; 1, 1–3 metastases all < 3 cm maximum diameter; 2, 4–19 metastases all < 3 cm maximum diameter or 1–3 metastases if any > 3 cm maximum diameter; 3, > 20 metastases any size or 4–19 metastases if any > 3 cm maximum diameter.

**Table VI** Other definitions of poor prognosis, advanced or bulky NSGCT

| |
|---|
| 1. Daugaard & Rorth (1986)<br>>10 cm diameter abdominal mass; liver metastases; supradiaphragmatic lymph node metastasis >5 cm diameter; multiple lung metastases with at least one >5 cm diameter; serum hCG >100,000 IU l$^{-1}$; extragonadal primary with raised markers. |
| 2. Ozols (1987)<br>>10 cm diameter abdominal or palpable mass; mediastinal mass; pulmonary nodule >5 cm diameter; >5 metastases per lung field with at least one >1 cm diameter; multiple lung nodules (>5 total) with at least one >2 cm diameter; pleural effusion; hypoxia ($pO_2$ <75 mmHg); pure choriocarcinoma histology; extragonadal primary; central nervous system involvement; liver or other visceral metastases; aFP>1,000 kU l$^{-1}$; hCG>10,000 IU l$^{-1}$. |
| 3. Schmoll et al. (1987)<br>>10 cm diameter abdominal mass; >5 lung metastases with diameter >2 cm each; >20 lung metastases with diameter <2 cm each; mediastinal mass >5 cm diameter; visceral, bone or central nervous system metastases. |
| 4. Pizzocaro et al. (1985)<br>>10 cm diameter abdominal mass, >5 cm diameter pulmonary nodule; liver, bone or central nervous system involvement; aFP>1,000 kU l$^{-1}$; hCG>50,000 IU l$^{-1}$. |

believe masses of this size are palpable readily in the NSGCT population consisting mainly of young non-obese males. Para-aortic node masses were palpable in patients in this series with radiological node dimensions less than 5 cm diameter.

Our patients who fulfilled the advanced disease, poor prognosis or bulky disease criteria of Daugaard & Rorth (1986), Ozols (1987) and Schmoll *et al.* (1987) were identified so outcome with these authors' regimens containing ultra-high dose DDP could be compared with POMB/ACE. Reclassification according to the far advanced disease criteria of Pizzocaro *et al.* (1985) was performed to allow comparison with a group receiving therapy based on conventional dose DDP (100 mg m$^{-2}$). All these criteria are shown in Table VI.

Survival curves were constructed by the method of Kaplan & Meier (1958) and compared by log rank analysis (Mantel, 1966).

### Results

Of the 206 patients, 13 were excluded from analysis because of (i) unknown initial disease parameters or incomplete staging (four patients), (ii) prior DDP-containing chemotherapy (six patients), and (iii) adjuvant chemotherapy administered after complete resection of metastatic disease in the presence of normal tumour markers (three patients).

The remaining 193 patients were fully assessable. Median age was 28 years (range 14–61). Histology (BTTP (18)) was malignant teratoma undifferentiated in 101 cases (52%), malignant teratoma intermediate in 53 (27%), malignant teratoma trophoblastic in 20 (10%), malignant teratoma differentiated in seven, seminoma in six and was unknown in six cases. Twenty patients (10%) were marker-negative but initial serum aFP was greater than 500 kU l$^{-1}$ in 35 cases (18%) and initial serum hCG greater than 50,000 IU l$^{-1}$ in 20 cases (10%) (Germa-Lluch *et al.*, 1980). Two patients with unknown histology but serum hCG in excess of 100,000 IU l$^{-1}$ at presentation were regarded as having trophoblastic histology under the EORTC criteria (Table V). All causes of death were included in subsequent survival analyses and, in particular, no early deaths were excluded. Twenty-two patients have died for an overall survival of 88.6% (171/193) at median follow-up 4.1 years (range 5 days to 8.7 years). The survival in the 20 patients with extragonadal primaries was not very different from the whole group: retroperitoneal primaries 7/12 (58%) and mediastinal primaries 8/8 (100%) giving an overall survival of 15/20 (75%). A specific and intractable problem in the retroperitoneal primaries was duodenal involvement which resulted in three of the deaths.

Figure 1 shows survival curves constructed after reclassification of our patients by MDA criteria, Figure 2 IND criteria, Figure 3 RMH criteria, Figure 4 MRC criteria, Figure 5 EORTC criteria and Figure 6 by CXH tumour marker criteria. Separation into MDA advanced disease ($P<0.02$ compared with minimal) and CXH poor prognosis ($P<0.001$ compared with good) groups was statistically significant by log rank analysis of survival curves. By IND classification, there was no statistical difference between minimal and moderate ($P>0.10$) or between moderate and advanced categories ($P>0.10$). However, if patients in IND minimal and moderate categories were combined then compared with those with IND advanced disease, significant separation of survival curves occurred ($P<0.005$).

Division of our patients into small and large volume groups by RMH criteria also resulted in significantly different survival curves ($P<0.02$). Reclassification by MRC criteria produced a well defined low risk group ($P<0.01$ low compared with intermediate risk, $P<0.001$ low compared with high risk) but no statistical difference was evident between intermediate and high risk groups ($P>0.10$). However, if high risk patients were compared with the remainder, the difference was significant ($P<0.05$). Allocation of our patients into four EORTC groups resulted in similar survival curves for the first three categories but significantly worse outcome in the fourth ($P<0.02$). All curves showed similar separation if 16 patients who had

**Figure 1** Survival curves after reclassification by MDA criteria into minimal ($n=77$) and advanced ($n=116$) disease.

**Figure 2** Survival curves after reclassification by IND criteria into minimal ($n=79$), moderate ($n=43$) and advanced ($n=71$) disease.

**Figure 3** Survival curves after reclassification by RMH criteria into small ($n=87$) and large ($n=106$) volume disease.

**Figure 4** Survival curves after reclassification by MRC criteria into low ($n=72$), medium ($n=67$) and high ($n=54$) risk disease. n.s., no significant difference (log rank).

**Figure 5** Survival curves after reclassification by EORTC criteria into four prognostic groups (1–4). n.s., no statistically significant difference (log rank).

received prior radiotherapy were excluded from analysis, although survival in each category was slightly better.

Figure 7 shows survival curves obtained when our patients with advanced, poor prognosis, bulky or far advanced disease defined by the criteria of Daugaard & Rorth (1986), Ozols (1987), Schmoll *et al.* (1987) and Pizzocaro *et al.* (1985) were compared. Table VII shows those authors' treatment regimens and published results in comparison with survival among our similarly defined adverse prognosis patients treated with POMB/ACE at same duration of follow-up.

**Figure 6** Survival curves after reclassification by CXH tumour marker criteria into good ($n=135$) and poor ($n=58$) prognosis disease.

**Figure 7** Survival curves of adverse prognosis patients according to the definitions of (a) Ozols (1987) ($n=113$), (b) Pizzocaro *et al.* (1985) ($n=75$), (c) Schmoll *et al.* (1987) ($n=67$) and (d) Daugaard & Rorth (1986) ($n=67$). n.s., no statistically significant difference (log rank).

Among 22 deaths in our series, the majority were recorded in the most advanced groups under each classification system or in the poor prognostic categories defined previously. However, the size of these 'adverse prognosis' groups ranged from 54 patients including 11 deaths (MRC high risk) to 116 patients including 19 deaths (MDA advanced). Therefore, survival data were adjusted for group size with true positive (TP) in each case regarded as the fraction dying in the adverse prognosis group; true negative (TN) the fraction alive in the favourable prognosis (remaining patients) group; false positive (FP) the fraction alive in the adverse prognosis group; and false negative (FN) the fraction dying in the favourable prognosis group. Sensitivity and specificity of each adverse prognosis definition in predicting death were calculated (sensitivity = TP/(TP + FN) x 100%; specificity = TN/(FP + TN) x 100%). Results are shown in Table VIII. Sensitivity varied by no more than 10% (range 71–81%) and specificity by less than 5% (range 52–56%) regardless of definition of adverse prognosis and were not altered significantly if deaths from intercurrent disease were excluded.

Seven deaths (32%) were noted two years or more after commencement of first-line chemotherapy and four of these fell into adverse prognostic groups by all definitions. A fifth patient was poor prognosis by CXH criteria but favourable prognosis under the other systems. The final two were both in the MDA advanced disease group. One patient died from myocardial infarction without evidence of residual NSGCT but all other late deaths occurred in patients with relapsed

**Table VII** Outcome after chemotherapy in poor prognosis, advanced or bulky NSGCT

| *Reference and regimen used* | *Overall survival* | *Median follow-up* | *CXH patients survival at same follow-up* |
|---|---|---|---|
| 1. Daugaard & Rorth (1986)<br>DDP 40 mg m$^{-2}$ × 5 days (3-weekly)<br>VP16 200 mg m$^{-2}$ × 5 days (3-weekly)<br>BLM 15 mg m$^{-2}$ weekly | 24/33 (73%) | 18.5 months | 59/67 (88%) |
| 2. Ozols (1987)<br>DDP 40 mg m$^{-2}$ × 5 days (3-weekly)<br>VP16 100 mg m$^{-2}$ × 5 days (3-weekly)<br>VLB 0.2 mg kg$^{-1}$ (3-weekly)<br>BLM 30 mg weekly | 23/30 (77%) | 27 months | 99/113 (88%) |
| 3. Schmoll *et al.* (1987)<br>DDP 35 mg m$^{-2}$ × 5 days (3-weekly)<br>VP16 120 mg m$^{-2}$ × 5 days (3 weekly)<br>BLM 15 mg m$^{-2}$ weekly | 69/98 (70%) | 2.2 years | 59/67 (88%) |
| 4. Pizzocaro *et al.* (1985)<br>DDP 20 mg m$^{-2}$ × 5 days (3-weekly)<br>VP16 100 mg m$^{-2}$ × 5 days (3-weekly)<br>BLM 18 mg m$^{-2}$ weekly | 34/40 (85%) | 2 years | 63/75 (84%) |

**Table VIII** Sensitivity and specificity of various adverse prognosis definitions in predicting death in patients with NSGCT

| *Criteria for adverse prognosis* | *Sensitivity* | *specificity* |
|---|---|---|
| MDA | 81% | 53% |
| IND | 79% | 54% |
| RMH | 79% | 53% |
| MRC | 73% | 54% |
| EORTC | 76% | 56% |
| CXH | 80% | 55% |
| Daugaard & Rorth (1986) | 77% | 54% |
| Ozols (1987) | 71% | 52% |
| Schmoll *et al.* (1987) | 80% | 55% |
| Pizzocaro *et al.* (1985) | 77% | 54% |

(four) or persistent (two) NSGCT. Two of four relapsed patients sustained their disease recurrence more than 12 months after completing first-line treatment in complete remission. The two patients never rendered completely disease-free had unresectable mature cystic teratoma in the retroperitoneum. Active tumour, including cerebral metastases, recurred in the first of these patients two years after completion of first-line therapy and caused his demise. The second patient's disease was controlled partially by various treatments over a long period but he developed acute non-lymphoblastic leukaemia and died more than 8 years after the original diagnosis of metastatic NSGCT.

### Discussion

These results imply that a poor prognostic category can be defined in metastatic NSGCT by initial serum marker (aFP, hCG) levels alone at least as reliably as by more complicated staging systems based on clinical and radiological measurement of disease extent. The method is easy to use in clinical practice and, because these markers reflect presence of the NSGCT components of greatest lethal potential (yolk sac, aFP; trophoblast, hCG (Paradinas, 1983), it is logical as well. Long-term survival of 100% among our marker-negative patients lends further support to this conclusion. Multiple combinations and permutations of published adverse prognostic criteria (Einhorn *et al.*, 1985; Newlands *et al.*, 1986; Logothetis *et al.*, 1986; Bosl *et al.*, 1986; Daugaard & Rorth, 1986; Ozols, 1987; Schmoll *et al.*, 1987; Medical Research Council Working Party on Testicular Tumours, 1985), only some of which are described in this paper, attest to the lack of agreement on definition of advanced, bulky or poor prognosis disease.

Chronologically, NSGCT deaths fall into two broad groups: (a) early, from overwhelming and rapidly progressive tumour at presentation; and (b) late, due to chemotherapy resistance. Our data suggest all commonly used definitions of adverse prognosis to have similar relatively modest sensitivity and specificity in predicting long-term outcome although early and late deaths were predicted equally. Long-term survival must remain the ultimate criterion of successful therapy in a highly responsive malignancy regardless of reported improvements in response rate. Numerically larger adverse prognostic groups (e.g. MDA: Ozols, 1987) included patients classified as good prognosis by other criteria, causing apparent 'dilution' of results.

Late death remains a problem in NSGCT, especially among poor prognosis patients. Many published series with average follow-up of two to three years cannot hope to address this (Williams *et al.*, 1987; Logothetis *et al.*, 1986; Daugaard & Rorth, 1986; Ozols, 1987; Schmoll *et al.*, 1987). Seven of 22 deaths (32%) in our series were noted two years or more from initiation of therapy and over half (12/22, 55%) occurred at more than 18 months. However, overall survival among all our patients (88.6%) is at least as good as any other published series and median follow-up is longer (4.1 years) than most. Late relapses and disease-related deaths after 5 years are also reported in long-term follow-up of PVB (cisplatin, vinblastine, bleomycin) treated patients from Indiana University (Greist *et al.*, 1985). Most of our late deaths were tumour-related although intercurrent causes were seen as well as one fatality from probable treatment-induced leukaemia. When we analysed these patients we found that reduced rates of drug delivery during first-line therapy were associated with subsequent relapse (Crawford *et al.*, 1988). Hence, intensifying frequency of drug administration in conventional dose regimens is a potential method for improving survival among adverse prognosis patients without resorting to high dose chemotherapy and subjecting the patients to its extra toxicity.

Treatment results in adverse prognosis NSGCT must be interpreted critically for the method of reporting results (response rates rather than overall survival) as well as duration of follow-up. Encouraging early results with new and more toxic treatment regimens in advanced disease categories may yield no better long-term outcome than conventional therapies. Improved response rate and survival in NSGCT have been demonstrated in a randomised study with combination chemotherapy using DDP doses of

120 mg m$^{-2}$ opposed to 75 mg m$^{-2}$ (Samson *et al.*, 1984). The use of ultra-high dose DDP (175–200 mg m$^{-2}$ per course) and VP16 regimens cause significant numbers of toxic deaths (Ozols, 1987; Schmoll *et al.*, 1987) and survival actually appears worse than the results with POMB/ACE (120 mg m$^{-2}$ DDP per course) (see Table IV). The results of Pizzocaro *et al.* (1985) with conventional doses of DDP and VP16 are similar to our own.

Bosl *et al.* (1983) performed a multivariate analysis of prognostic variables which identified the concentration of lactate dehydrogenase, hCG and the total number of metastases as the major adverse prognostic variables. They used these variables to identify a group of patients who received alternating chemotherapy in an attempt to improve their survival. However, although this was not a randomised comparison there was no improvement in the results with their approach (Bosl *et al.*, 1987). More recently the same group have performed a parallel analysis to that reported here (Bajorin *et al.*, 1988). In a smaller series of patients with a shorter follow-up they have also identified the limitations of current staging classifications. They found that there were differences in sensitivity, specificity and predictive value in the classifications they analysed. They included two systems analysed in this report (IND and EORTC) and also reported on their own system (Memorial Sloan-Kettering Cancer Center (MSKCC)) and the National Cancer Institute (NCI) systems. We agree with their conclusions that randomised trials with agreed eligibility criteria for entry will be required before conclusions on the relative efficacy of different regimens of chemotherapy can be made.

This work was supported by the Cancer Research Campaign.

### References

BAJORIN, D., KATZ, A., CHAN, E. & 3 others (1988). Comparison of criteria for assigning germ cell tumor patients to 'good risk' and 'poor risk' studies. *J. Clin. Oncol.*, **6**, 786.

BEGENT, R.H.J. & BAGSHAWE, K.D. (1983). Staging, markers, and prognostic factors. In *Clinics in Oncology 2(1) – Germ Cell Tumours*, Bagshawe, K.D., Newlands, E.S. & Begent, R.H.J. (eds) p. 159. Saunders: London.

BIRCH, R., WILLIAMS, S.D., CONE, A. & 4 others (1986). Prognostic factors for favorable outcome in disseminated germ cell tumours. *J. Clin. Oncol.*, **4**, 400.

BOSL, G.J., GELLER, N.L., CIRRINCIONE, C. & 7 others (1983). Multivariate analysis of prognostic variables in patients with metastatic testicular cancer. *Cancer Res.*, **43**, 3403.

BOSL, G.J., GELLER, N.L., VOGELZANG, N.J. & 7 others (1987). Alternating cycles of etoposide plus cisplatin and VAB-6 in the treatment of poor-risk patients with germ cell tumours.

BOSL, G.J., GLUCKMAN, R., GELLER, N.L. & 8 others (1986). VAB-6 – an effective chemotherapy regimen for patients with germ cell tumours. *J. Clin. Oncol.*, **4**, 1493.

CRAWFORD, S.M., NEWLANDS, E.S., BEGENT, R.H.J., RUSTIN, G.J.S. & BAGSHAWE, K.D. (1988). The effect of intensity of administered treatment on the outcome in germ cell tumours treated with POMB/ACE chemotherapy. (Submitted for publication).

DAUGAARD, G. & RORTH, M. (1986). High-dose cisplatin and VP-16 with bleomycin in the management of advanced germ cell tumours. *Eur. J. Cancer Clin. Oncol.*, **22**, 477.

EINHORN, L.H., DONOHUE, J.P., PECKHAM, M.J. & 2 others (1985). Cancer of the testes. In *Cancer – Principles and Practice of Oncology*, 2nd edn, DeVita, V.T., Hellman, S. & Rosenberg, S.T. (eds) p. 979. Lippincott: Philadelphia.

EINHORN, L.H. (1986). Have new aggressive chemotherapy regimens improved results in advanced germ cell tumours? *Eur. J. Cancer Clin. Oncol.*, **22**, 1289.

GERMA-LLUCH, J.R., BERGENT, R.H.J. & BAGSHAWE, K.D. (1980). Tumour marker levels and prognosis in malignant teratoma of the testis. *Br. J. Cancer*, **42**, 850.

GREIST, A., ROTH, B., EINHORN, L. & WILLIAMS, S.D. (1985). Cisplatin-combination chemotherapy for disseminated germ cell tumours: long term follow-up (abstract). *Proc. Am. Soc. Clin. Oncol.*, **4**, 100.

KAPLAN, E.L. & MEIER, P. (1958). Nonparametric estimation from incomplete observations. *J. Am. Stat. Assoc.*, **53**, 457.

LOGOTHETIS, C.J., SAMUELS, M.L., SELIG, D.E. & 5 others (1986). Cyclic chemotherapy with cyclophosphamide, doxorubicin, and cisplatin plus vinblastine and bleomycin in germinal tumours – results with 100 patients. *Am J. Med.*, **81**, 219.

MANTEL, N. (1966). Evaluation of survival data and two new rank order statistics arising in its consideration. *Cancer Chemother. Rep.*, **50**, 163.

MEDICAL RESEARCH COUNCIL WORKING PARTY ON TESTICULAR TUMOURS (1985). Prognostic factors in advanced non-seminomatous germ-cell testicular tumours – results of a multi-centre study. *Lancet*, **i**, 8.

NEWLANDS, E.S., BAGSHAWE, K.D., BEGENT, R.H.J. and 3 others (1986). Current optimum management of anaplastic germ cell tumours of the testis and other sites. *Br. J. Urol.*, **58**, 307.

OZOLS, R.F. (1987). Treatment of poor prognosis germ cell tumours with high dose cisplatin regimens. *Int. J. Androl.*, **10**, 291.

PARADINAS, F. (1983). Pathology. In Clinics in Oncology 2(1) – Germ Cell Tumours. Bagshawe, K.D., Newlands, E.S. & Begent, R.H.J. (eds) p. 17. Saunders: London.

PECKHAM, M.J. (1981). Investigation and staging – general aspects and staging classifications. In *The Management of Testicular Tumours*. Peckham M.J. (ed.) p. 89. Arnold: London.

PIZZOCARO, G., PIVA, L., SALVIONI, R., ZANONI, F. & MILANI, A. (1985). Cisplatin, etoposide, bleomycin as first-line therapy and early resection of residual tumour in far advanced germinal testis cancer. *Cancer*, **56**, 2411.

PUGH, R.C.B. (1976). Testicular tumours – the panel classification. In *Pathology of the Testis*, Pugh, R.C.B. (ed.) p. 144. Blackwell Scientific: London.

SAMSON, M.K., RIVKIN, S.E., JONES, S.E. & 5 others (1984). Dose-response and dose-survival advantage for high versus low dose cisplatin combined with vinblastine and bleomycin in disseminated testicular cancer. *Cancer*, **53**, 1029.

SCHMOLL, H.J., SCHUBERT, I., ARNOLD, H. & 5 others (1987). Disseminated testicular cancer with bulky disease: results of a phase-II study with cisplatin ultra high dose/VP16/bleomycin. *Int. J. Abdrol.*, **10**, 311.

STOTER, G., SYLVESTER, R., SLEIFER, D.T. & 7 others (1987). Multivariate analysis of prognostic factors in patients with disseminated nonseminomatous testicular cancer – results from a European Organization for Research on Treatment of Cancer multi-institutional phase III study. *Cancer Res.*, **47**, 2714.

VIGRIN, D., GRIEDMAN, A. & WHITMORE, W.F. (1984). Correlation of serum tumour markers in advanced germ cell tumours with responses to chemotherapy and surgery. *Cancer*, **53**, 1440.

WILLIAMS, S.D., BIRCH, R., EINHORN, L.H. & 3 others (1987). Treatment of disseminated germ-cell tumours with cisplatin, bleomycin, and either vinblastine or etoposide. *N. Engl. J. Med.*, **316**, 1435.

### Appendix

*POMB/ACE chemotherapy schedules*

These chemotherapy schedules have been reported previously (Newlands *et al.*, 1986).

*POMB*

Day 1 Vincristine 1 mg m$^{-2}$ intravenously; methotrexate 300 mg m$^{-2}$ as a 12 h infusion.

Day 2 Bleomycin 15 mg as a 24 h infusion; folinic acid rescue started at 24 h after the start of methotrexate in a dose of 15 mg 12-hourly for four doses.

Day 3 Bleomycin infusion 15 mg by 24 h infusion

Day 4 Cisplatin 12 mg m$^{-2}$ as a 12 h infusion, given together with hydration and 3 g magnesium sulphate supplementation.

*ACE*

Etoposide (VP 16-213) $100\,mg\,m^{-2}$, days 1–5; actinomycin D 0.5 mg i.v. days 3, 4 and 5; cyclophosphamide $500\,mg\,m^{-2}$ i.v., day 5.

*OMB*

Day 1 Vincristine $1\,mg\,m^{-2}$ intravenously, methotrexate $300\,mg\,m^{-2}$ as a 12 h infusion.

Day 2 Bleomycin 15 mg by 24 h infusion: folinic acid rescue started at 24 h (after the start of methotrexate) in a dose of 15 mg 12-hourly for four doses.

Day 3 Bleomycin 15 mg by 24 h infusion.

The sequence of treatment schedules is two courses of POMB followed by ACE. POMB is then alternated with ACE until patients are in biochemical remission as measured by hCG and aFP (and, if raised, lactate dehydrogenase). The usual number of courses of POMB has been three to five. Following biochemical remission, patients alternate ACE with OMB until remission has been maintained for approximately 12 weeks. The intervals between each course of treatment have been kept to the minimum (usually 9–11 days). If delays are caused by myelosuppression following courses of ACE, the first two days of etoposide are omitted from subsequent courses of ACE. Unless there are delays patients receive $360\,mg\,m^{-2}$ of cisplatinum in the first 8 weeks of therapy.

*Central nervous system metastases*

*Prophylaxis.* Most patients had pulmonary metastases at presentation. Our current policy is to give CNS prophylaxis with injections of intrathecal methotrexate 12.5 mg with the first three courses of chemotherapy if the patients have pulmonary metastases and/or an initial serum hCG $> 1{,}000\,IU\,l^{-1}$. Cerebrospinal fluid (CSF) concentrations of hCG and aFP were measured routinely.

*Established CNS metastases.* The dose of methotrexate with each course of POMB is increased to $1\,g\,m^{-2}$ and infused over 24 h. Folinic acid rescue starts 8 h later in a dose of 15 mg 6-hourly for 72 h. Intrathecal methotrexate in a dose of 12.5 mg is given with the courses of ACE and continued until the brain metatases have completely regressed as measured by CT scanning and CSF HCG concentrations.

*Dosage reduction in patients presenting with respiratory, liver or renal failure.* Rapid improvements can be obtained in these very sick patients using a combination of etoposide $100\,mg\,m^{-2}$ i.v. and cisplatin (EP) $20\,mg\,m^{-2}$ i.v. on days 1 ans 2 (this can be increased to 3 days as indicated). These courses are repeated at short intervals (5–7 drug free days) and normally the full schedule with POMB/ACE can be started after two or three courses of EP.